# Intraparotid lymph node metastasis affects distant metastasis in parotid adenoid cystic carcinoma

**DOI:** 10.1038/s41598-023-38227-1

**Published:** 2023-07-11

**Authors:** Xiaoxue Han, Jia Wang, Yuexiao Li, Shanlong Xi, Weiwei Xiao

**Affiliations:** 1 Department of Oral Maxillofacial Surgery, Affiliated Stomatology Hospital of China Medical University, Shenyang, Liaoning People’s Republic of China; 2 Department of Thoracic Surgery, The First Affiliated Hospital of China Medical University, Shenyang, Liaoning People’s Republic of China

**Keywords:** Head and neck cancer, Oral cancer, Surgical oncology

## Abstract

To evaluate the relationship between factors of metastatic intraparotid lymph node (IPLN) and distant metastasis in parotid adenoid cystic carcinoma (ACC). Patients with surgically treated parotid ACC were retrospectively enrolled, and primary outcome variable was distant metastasis free survival (DMFS). The effect of factors of metastatic IPLN on DMFS was evaluated using Cox model. In total, 232 patients were included. Extranodal extension of IPLN and cervical lymph nodes did not impact the DMFS, and the 7th but not 8th AJCC N stage was associated with DMFS. Groups of 0 and 1 metastatic IPLN had comparable DMFS, but presence of 2+ positive IPLN was related to increased worse DMFS (p = 0.034, HR 2.09). A new N stage (0 vs 1–2 vs 3+) based on total positive lymph node number exhibited better C-index than traditional N stage. IPLN metastasis increased the risk of distant metastasis, and the impact was mainly determined by the number of metastatic IPLN. Our proposed N stage provided better DMFS prediction than the 8th AJCC N classification.

## Introduction

Salivary gland carcinomas are relatively uncommon, and account for less than 3% of all head and neck cancers^1^, most of them occur in parotid gland. One of the most frequent pathologic types is adenoid cystic carcinoma (ACC), it is featured by distant metastasis (DM)^2^. Complete resection is the preferred method, but DM is likely to develop in 40% or more of the patients and also the main cause of death during follow-up^3^. To detect the potential predictors for DM carries essential significance to improve the oncologic outcome by filtering high risk patients.

At present, prior evidence has described that perineural invasion (PNI), lymphovascular invasion (LVI), cervical lymph node (LN) metastasis, and positive margin increase the risk of DM^4–9^, but the role of intraparotid lymph node (IPLN) is rarely discussed, to our best knowledge, only one research has reported the association between IPLN metastasis and DM in parotid cancer^10^, in this study, positive IPLNs were noted in 31.8% of the sample and provided nearly additional onefold possibility of DM compared with non-metastasis group in Cox model analysis. But there is still much unknown regarding how burden and extranodal extension (ENE) of metastatic IPLNs impact the DM risk.

Therefore, the goal of current study was to evaluate the relationship between factors of metastatic IPLN and DM in parotid ACC.

## Patients and methods

### Ethical consideration

This study was approved by China Medical University Institutional Research Committee, all methods were performed in accordance with the relevant guidelines and regulations, and written consent agreements for medical research were obtained from all patients before the initial treatment.

### Study design

The investigators performed a retrospective study to address the issue. From January 2000 and December 2022, medical records of patients with surgically treated parotid ACC were reviewed. Inclusion criteria were presented as follows: the disease was primary; pathologic section was available for re-reviewing; follow-up data could be obtained; neck dissection or sentinel pathologic examination of at least four cervical LNs were conducted^11^. Patients with a history of other malignancy or DM at initial treatment were excluded. Information regarding demography, pathology, treatment and follow-up of included patients was extracted.

### Study variable

All pathologic sections were re-reviewed by at least two head and neck pathologists to confirm the correct diagnosis of ACC. IPLN referred to the LNs located within the parotid gland. Tumor stage was formulated based on the 8th AJCC system, neck classification were formulated based on the 7th and 8th AJCC system, pathologic grade was defined as low for tubular or cribriform type, in which a solid component accounted for less than 30%, and high for solid type^12^. PNI was defined as positive if there were tumor cells within the nerve, LVI was defined as positive if there were tumor cells within the lymphovascular vessel, ENE was defined as positive if there were tumor cells outside the capsule of metastatic LN. Total number of positive LNs was defined as the sum of the number of positive IPLNs and cervical LNs.

The primary outcome variable was distant metastasis free survival (DMFS). It was confirmed via biopsy or image analysis if there was difficult in puncture^13^. Its time was calculated from the date of surgery to the date of DM detection or the last follow-up. For all patients after treatment completion, they were followed up every 3–6 months for the first two years, every 6–12 months for the next three years, and every 12–24 months thereafter.

### Treatment principle

Intraoperative pathologic examination of cervical LNs was routinely performed if frozen section of primary tumor indicated a malignancy^11^. Dissected LNs consisted of level I–IV/V if neck dissection was performed, and postoperative radiotherapy was suggested if there was presence of ACC independent of other adverse pathologic features. Adjuvant chemotherapy was decided on the physician’s experience and presence of positive margin and ENE of cervical LN.

### Statistic analysis

The Kaplan–Meier method was used to compare the DMFS rates in patients with different factors of IPLN, and the factors which were significant in univariate analyses were then evaluated in Cox proportional hazard regression analyses to determine the independent predictors. Three Cox models were constructed and compared using C-index, a higher C-index meant better prognosis prediction, number of positive IPLNs was analyzed in model 1, the 7th AJCC N stage and number of positive IPLNs were analyzed in model 2, total number of positive LNs was analyzed in model 3. All analyses were manipulated using R 3.4.3, and a p less than 0.05 was considered to be significant.

### Ethics approval and consent to participate

This study was approved by China Medical University Institutional Research Committee, and written consent agreements for medical research were obtained from all patients before the initial treatment.

## Results

### Baseline data

In total, 232 patients were included with a mean age of 53 ± 18 years, there were 101 (43.5%) male and 131 (56.5%) female. Pathologic tumor stages were classified as T1 in 70 (30.2%) patients, T2 in 90 (38.8%) patients, T3 in 50 (21.6%) patients, and T4 in 22 (9.5%) patients. Pathologic grade was low in 121 (52.2%) patients and high in 111 (47.8%) patients. Pathologic neck stage was N0 in 194 (83.6%) patients, of whom 87 necks were staged based on sentinel biopsy and 107 necks were staged according to lymphadenectomy, in the rest 38 patients, ENE developed in 5 patients, N1 was in 25 (10.8%) cases, N2 was in 11 (4.7%) cases, and N3 was in 2 (0.9%) cases. PNI occurred in 49 (21.1%) patients, and LVI (18.5%) showed in 43 patients. Positive margin developed in 42 (18.1%) patients. IPLN metastasis presented in 46 (19.8%) patients, of whom 28 cases had one positive LN, 15 cases had two, and 3 cases had three. ENE developed in 17 (37.0%) of the 46 patients. In patients without IPLN metastasis, the median number of examined IPLNs was 2 (range 1 to 6). Total number of metastatic LNs was 0 in 170 (73.3%) patients, 1 in 25 (10.8%) patients, 2 in 18 (7.8%) patients, 3 in 12 (5.2%) patients, 4 in 4 (1.7%) patients, and 5 in 3 (1.3%) patients. A total of 189 patients underwent adjuvant radiotherapy, in those patients, the median radiation dose was 56 Gy (range 46–66 Gy), and the area of irradiation included primary site and ipsilateral level I to IV/V, adjuvant chemotherapy was performed in 60 patients.

### Association between IPLN metastasis and neck stage

In Table 1, in 219 patients with a N0 or N1 neck, 25 cases had one positive IPLN, and 10 cases had two positive LNs, in the rest 13 patients with a N2/3 neck, 5 cases had two metastatic IPLNs, and 3 cases had three metastatic LNs, the difference was significant (p < 0.001, Table 1).

**Table 1:** Association between intraparotid lymph node metastasis and neck stage.

Neck stage	Number of metastatic intraparotid lymph node				p
	0 (n = 186)	1 (n = 28)	2 (n = 15)	3 (n = 3)	
N0 (n = 194)	171	17	6	0	
N1 (n = 25)	13	8	4	0	
N2 (n = 11)	2	3	4	2	
N3 (n = 2)	0	0	1	1	< 0.001

### Predictors for DM

During our follow-up with mean time of 4.2 ± 2.5 years, distant metastasis occurred in 125 (53.9%) patients, and the mean time of DM development was 3.1 ± 1.5 years, the overall 5-year DMFS rate was 46% (95% CI 38–54%). Lung was the most common metastasized site and developed in 100 patients, of whom 74 patients had only lung distant and 26 patients had other metastasis sites simultaneously (bone: 14 cases; liver: 12 cases; brain: 5 cases; renicapsule: 3 cases; skeletal muscle: 1 case). In the rest 25 patients, bone metastasis occurred in 12 patients, liver metastasis occurred in 10 patients, and brain metastasis occurred in 6 patients.

**Figure 1:**
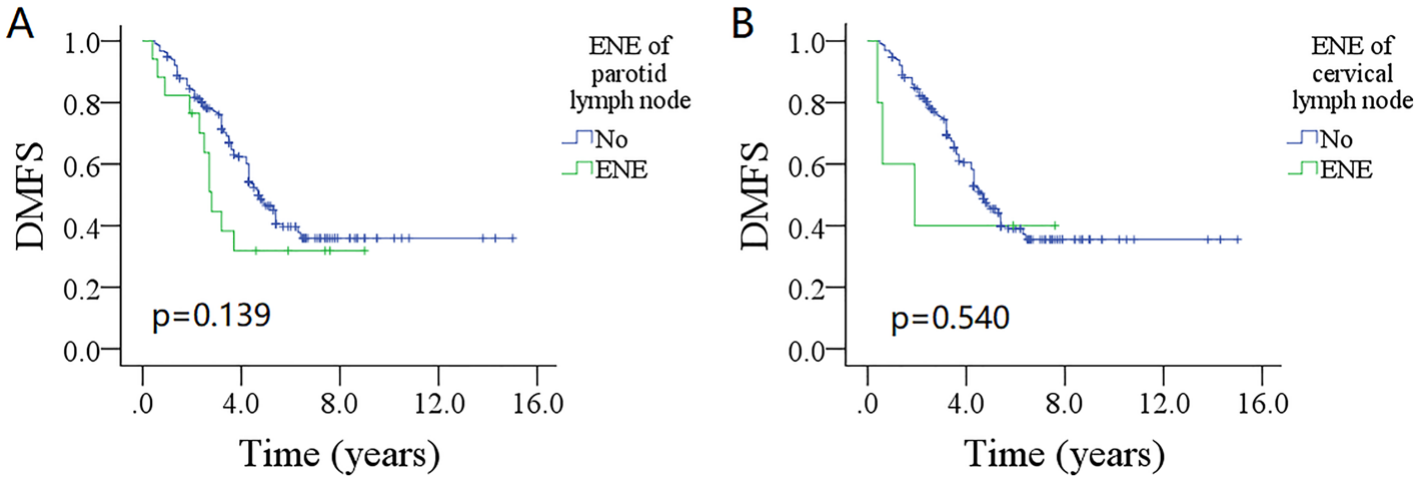
Comparison of distant metastasis free survival (DMFS) between patients with different status of extranodal extension (ENE); (**A**) for ENE in intraparotid lymph node (p = 0.139), (**B**) for ENE in cervical lymph node (p = 0.540).

**Figure 2:**
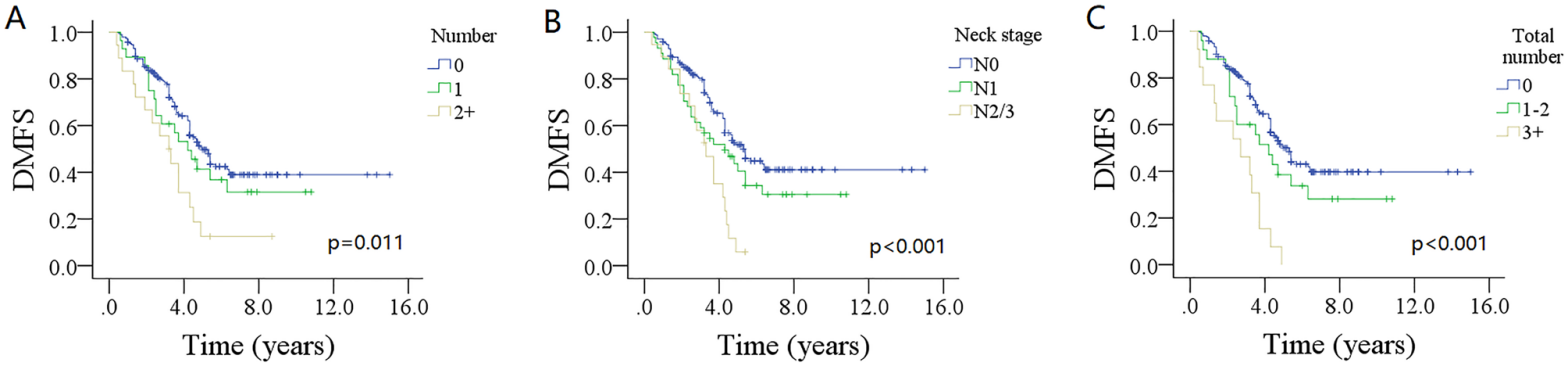
Comparison of distant metastasis free survival (DMFS) among different prognostic models; (**A**) for number of positive intraparotid lymph node (p = 0.011), (**B**) for the 7th AJCC neck stage (p < 0.001), (**C**) for total number of positive lymph nodes (p < 0.001).

In univariate analysis, age, sex, LVI, ENE of positive IPLN and cervical LNs (Fig. 1A,B), the 8th neck stage, and adjuvant therapy did not impact the DMFS (all p > 0.05). But tumor stage, number of metastatic IPLNs (Fig. 2A), the 7th neck stage (Fig. 2B), pathologic grade, PNI, positive margin, and total number of positive LNs (Fig. 2C) was related to DM (Table 2).

**Table 2:** Univariate analysis of predictors for distant metastasis free survival.

Variable	Univariate analysis
Age	
< 50	
≥ 50	0.327
Sex	
Male	
Female	0.522
Tumor stage	
T1	
T2	
T3	
T4	< 0.001
7th Neck stage	
N0	
N1	
N2 + N3	< 0.001
8th Neck stage	
N0	
N1	
N2 + N3	0.104
Pathologic grade	
Low	
High	< 0.001
Perineural invasion	0.005
Lymphovascular invasion	0.142
Positive margin	< 0.001
Number of metastatic intraparotid lymph nodes	
0	
1	
2+	0.011
Total number of metastatic lymph nodes	
0	
1–2	
3+	< 0.001
Extranodal extension of intraparotid lymph node	0.139
Extranodal extension of cervical lymph node	0.54
Treatment	
Surgery	
Surgery + adjuvant radiotherapy	
Surgery + adjuvant chemoradiotherapy	0.332

The 5-year DMFS rate was 46% (95% CI 38–54%) for patients with no IPLN metastasis, 64% (95% CI 46–82%) for patients with 1 positive LN, 13% (95% CI 0–29%) for patients with 2 or more positive LNs, the difference was significant (p = 0.011). The 5-year DMFS rates were 47% (95% CI 39–55%) for patients without ENE in IPLN, and 33% (95% CI 9–57%) for ENE group, the difference was not significant (p = 0.139). The 5-year DMFS rate was 48% (95% CI 44–52%) for patients with none positive LN, 40% (95% CI 34–46%) for patients with 1/2 positive LNs, and 8% (0–16%) for patients with 3 or more positive LNs, the difference was significant (p < 0.001).

Model 1 included tumor stage, pathologic grade, PNI, positive margin, and number of metastatic IPLNs. Zero and one positive IPLN groups had comparable DM risk, but presence of 2 or more metastatic IPLNs was related to about twofold risk (95% CI 1.18–5.34) of DM. Other independent factors included T4 stage, high grade, and positive margin (Table 3). The C-index was 0.69 (95% CI 0.65–0.73).

**Table 3:** Cox model analyses of predictors for distant metastasis free survival.

Variable	Model 1		Model 2		Model 3	
	HR	95% CI	HR	95% CI	HR	95% CI
T						
T1		Ref		Ref		Ref
T2	0.533	1.90 [0.68–3.22]	0.145	2.03 [0.75–4.38]	0.032	1.68 [1.04–3.63]
T3	0.081	2.01 [0.93–4.19]	0.021	2.76 [1.25–6.95]	0.004	2.35 [1.46–6.62]
T4	< 0.001	3.47 [1.24–7.73]	0.001	3.63 [1.84–10.54]	< 0.001	3.24 [1.90–9.73]
7th N stage						
N0		–		Ref		–
N1		–	0.103	1.95 [0.87–4.36]		–
N2 + N3		–	0.021	3.21 [1.76–8.41]		–
Pathologic grade						
Low				Ref		Ref
High	< 0.001	3.90 [1.74–17.88]	< 0.001	5.47 [2.17–20.73]	< 0.001	4.37 [2.05–18.75]
Perineural invasion	0.178	2.18 [0.73–8.61]	0.261	2.07 [0.53–6.42]	0.016	1.97 [1.28–6.96]
Positive margin	< 0.001	4.76 [2.08–10.33]	< 0.001	3.04 [1.67–9.44]	< 0.001	5.82 [2.74–17.84]
Number of metastatic intraparotid LNs						
0		Ref		Ref		–
1	0.567	1.75 [0.64–4.27]	0.205	1.85 [0.73–4.14]		–
2+	0.034	2.09 [1.18–5.34]	0.005	2.76 [1.26–6.48]		–
Total number of metastatic LNs						
0		–		–		Ref
1–2		–		–	< 0.001	2.15 [1.33–6.58]
3+		–		–	< 0.001	4.27 [2.36–18.29]

–The variable was not included in the corresponding model.

Model 2 included tumor stage, the 7th neck stage, pathologic grade, PNI, positive margin, and number of metastatic IPLNs. Compared with no IPLN metastasis group, presence of 1 positive LN did not impact the DM, but presence of 2 or more metastatic IPLNs was associated with nearly threefold risk (95% CI 1.26–6.48) of DM. N1 and N0 stages had comparable possibility of DM, but N2/3 stage predicted significantly higher risk of DM (HR: 3.21; 95% CI 1.76–8.41). Other independent factors included T3/4 stage, high grade, and positive margin (Table 3). The C-index was 0.74 (95% CI 0.72–0.76).

Model 3 included tumor stage, pathologic grade, PNI, positive margin, and total number of positive LNs. Compared with no LN metastasis, one or two positive LNs had a HR of 2.15 (95% CI 1.33–6.58), and 3 or more LNs meant the worst DMFS (HR 4.27; 95% CI 2.36–18.29) (Table 3). The C-index was 0.77 (95% CI 0.73–0.81).

## Discussion

Our most important finding was that IPLN metastasis increased the risk of DM, and the impact was mainly determined by the number rather than ENE of metastatic IPLNs. The 7th but not 8th AJCC N stage was related to DM development, prognostic model based on total number of metastatic LNs provided better survival stratification than those according to number of metastatic IPLNs and/or the AJCC N stage.

Metastatic IPLN was an important prognostic factor in parotid cancer, it was related to decreased survival^14,15^, but DMFS was analyzed as an outcome variable only in a few studies^10,16^, the risk of DM was significantly increased if there was presence of metastatic IPLN, and the HR ranged from 2 to 4, however, all these researches evaluated the IPLN status as a binary variable (Yes vs No), it remained unclear regarding the effect of positive IPLN burden on prognosis. Feng et al.^17^ might be the only one to evaluate the association between different positive LN number of parotid and survival in 337 patients, compared with no IPLN metastasis, 1 or 2 positive LNs linked with nearly sixfold risk of local recurrence, and 3 or more LNs had a HR of 21, but the authors failed to report the impact on DM. Our study firstly noted groups of 0 and 1 metastatic IPLN had similar DMFS, and presence of 2+ positive LNs predicted poorer control of DM. The finding posed meaningful clinical significance, because for most solid cancers, worse oncologic outcome would be expected even if there was only one metastatic LN, and aggressive treatments were likely to be given to these patients to improve the prognosis, such as adjuvant radiotherapy would be suggested for parotid ACC with no other adverse pathologic features but only one positive IPLN, our study may alter the senseless practice. The underlying mechanism could be explained by the relatively low LN metastasis frequency and slow-growing of parotid ACC.

Considering the significance of IPLN metastasis, a suggestion of the inclusion of IPLN status in LN stage classification was proposed^18^, but this was not realized in the 8th AJCC N classification which was drafted based on head and neck squamous cell carcinoma^19^, prior literature described the official N stage could not well stratify the survival in salivary gland carcinoma^20^, our study also confirmed that the 8th AJCC N stage was not related to DM occurrence. It might be contributed by the distinct difference of biologic behaviour between the two kinds of disease, and the common death cause in parotid cancer was DM but not locoregional recurrence. Another factor that could not be ignored was ENE, which was usually an indicator for adjuvant chemotherapy to oppose the high possibility of DM in head and neck cancer, but it was false in parotid ACC based on our results. In a study including 114 patients with pN + salivary gland carcinoma^21^, ENE developed in 51% of the cases, and was related to PNI, LVI, advanced N stage, and higher number of positive LNs, but had no association with demography, tumor stage origin, and histology grade. After adjusting the number of positive cervical LNs, ENE did not impact the survival. In another similar study^22^, ENE occurred in 27 (40.9%) patients, and ENE group had comparable locoregional-free survival, overall survival, and DMFS with those without ENE. The conclusion was also confirmed by Lombardi et al.^23^. These findings elucidated that ENE of cervical LN tended to demonstrate limited impact on prognosis, but was associated with some adverse pathologic features which drove the prognosis actually. The interesting discovery was also appropriate in ENE of IPLN, its presence did not add any supernumerary DMFS decrease, and it could explained the fact that the 7th but not 8th AJCC N stage was related to the DMFS, and the underlying mechanism for the firstly reported finding might be explained by the small anatomic size of IPLN, even a minimal lesion could easily break through the capsule.

An alternative LN stage was needed to better stratify the survival of parotid cancer. Aro et al.^20^ introduced a LN stage based on the metastatic LN number (0 vs. 1–2 vs. 3–21 vs. 22 +) after analyzing the outcomes in 4520 patients undergoing neck dissection for salivary gland carcinoma, it provided better survival prediction than the 8th AJCC N stage. Another three-category LN stage according to the number of positive LNs and ENE was also superior to the 8th AJCC N stage in prognostic calculation^24^. However, the impact of IPLN on survival was neglected in the two studies, and the variable was neither incorporated into the proposed N stages nor analyzed in a regression model. Very few authors had evaluated the IPLN and neck stage as one variable. In a study consisting of 307 patients treated for salivary gland carcinoma^23^, owing to the failure of the 8th AJCC classification in overall survival stratification, the authors described two new LN systems according to the number of positive LNs (0 vs 1–3 vs 4+) and/or their maximum diameter (< 20 mm vs 20+ mm) showed better accuracy in survival prediction. Boon et al.^25^ assessed the outcomes of 177 salivary duct carcinoma patients and noted that the absolute number of positive LNs (0 vs 1–2 vs 3–15 vs 16+), rather than the traditional cervical stage, was the only significant prognostic factor for overall survival in the multivariate analysis. It remained unknown whether such classification could apply for parotid ACC which had apparently different features with other parotid cancers. In current study, we formulated a three-category LN stage with combination of metastatic parotid and cervical LNs, the system had the highest C-index among the three models in predicting DMFS, it was simple and suitable for clinical use effectively^26^. But it was related to increased demands for LN detection, and detection of a small IPLN was usually time-consuming and labor-intensive, and required cooperation of surgeon and pathologists, sometimes the entire parotid gland should be microscopically examined for accurate diagnosis.

Limitation in current study must be acknowledged, first, this was a retrospective study, it had inherent bias; second, only ACC was analyzed, it was not clear whether the finding could be realized in other histologic types; third, this was a single institution study, before clinical application, further validation was required.

## Conclusion

In summary, IPLN metastasis increased the risk of DM, and the impact was mainly determined by the number rather than ENE of metastatic IPLNs. Our proposed N stage provided better DMFS prediction than the 8th AJCC N classification.

## Data Availability

All data generated or analyzed during this study are included in this published article. And the primary data could be achieved from the corresponding author.
